# A New Method of Treating Diphtheria

**Published:** 1886-01

**Authors:** 


### A New Method of Treating Diphtheria.

Dr. R. J. Nunn, of Savannah, Ga., in an article published in the *Atlanta Medical and Surgical Journal* for October, advocates a method of treating diphtheria, which deserves the attention of medical men. The following is an abstract of the article: "In the study of a successful mode of treating diphtheria, two aims must be kept constantly before the mind: 1, To neutralize the constitutional taint; 2, To remove, and arrest the formation of, local deposits.

Assuming that the cause of diphtheria is a microbe, the destruction of the germ may be accomplished in two ways:

1. By chemical re-agents, known as antiseptics, etc.
2. By the discovery of some other micro-organism which will eat up or destroy the microbe of diphtheria."

Neglecting the second method, the writer endeavors to show that we are in possession of germicides which can be introduced into the blood in sufficient quantities to destroy the microbes without affecting the vitality of the patient. These antiseptics are the biniodide of mercury and the iodide of potassium. According to the experiments of Dr. Miguel, one part of biniodide of mercury will render 40,000 parts of beef tea antiseptics.

Hence, one grain of the biniodide will render the body of a child weighing fifty pounds antiseptic. But it is manifestly impossible to give this drug in such quantities. Dr. Miguel found that the proportion of iodide of potassium required to sterilize beef tea was one-seventh, but one-ninth or even less of this quantity will render urine antiseptic. Dr. Nunn accordingly concludes that one-eighteenth of a grain of the biniodide of mercury will prevent the development of bacteria in a child weighing fifty pounds, and this minute proportion may be still further reduced by giving iodide of potassium with it. One of the first indications to be fulfilled is to keep the system saturated with the antiseptic, and, as these drugs are rapidly eliminated from the system, they must be given at short intervals. One grain of the biniodide is dissolved in from four to eight ounces of water, and iodide of potassium added according to the age and condition of the patient.

For combating local symptoms, the peroxide of hydrogen is preferred as a solvent of the membranes. This drug, while acting as a powerful solvent of the membrane, has no effect upon the healthy tissue; besides, this remedy cannot be classified with the poisons. The only drawback to the employment of this agent is, that, although it rapidly dissolves and washes away recent deposits, it is almost powerless against thickened and hardened masses; hence arises the necessity for a digestive agent. For this purpose, pepsin, pancreatin, trypsin and papayotin have been used. The latter is to be preferred, because it acts equally well in alkaline or acid solutions. Agavin is also suggested.

The papayotin is applied to the membrane by means of a powder blower, or it may be used in solution. The parts should first be cleaned with peroxide of hydrogen, and nothing should be given for half an hour afterward, the object being to avoid washing off the papayotin before it has time to act. This agent is to be applied two or three times daily, the ethereal solution of iodoform being employed also.

Dr. Nunn gives a resumè of his treatment as follows:

1. Give five or six drops of the constitutional antiseptic (solution of biniodide of mercury with iodide of potassium) every ten minutes. If the patient is asleep, the medicine may be dropped into the mouth with a French pipette or a camel's hair brush, without waking the child.

2. Twice, or oftener daily, wash off all membrane in throat, nasal passages, etc., with a solution of peroxide of hydrogen or other suitable solvent.

3. Apply to the membrane papayotin or some agent possessing similar properties.

4. Let the patient alone for half an hour.

5. Resume internal medicine.

6. In the interval, use ethereal solution of iodoform locally.

7. Stimulants and nutriments are to be pushed as the basis of all treatments.

8. As the case recovers gradually, the local applications are to be discontinued.

9. In like manner, the frequency of the doses of the constitutional antiseptic is to be lessened, but—

10. It should not be wholly discontinued until all danger of sequels is past.

